# Phytotoxic Potential and Phenolic Profile of Extracts from *Scrophularia striata*

**DOI:** 10.3390/plants10010135

**Published:** 2021-01-11

**Authors:** Seyyed Sasan Mousavi, Akbar Karami, Tahereh Movahhed Haghighi, Saeed Alizadeh, Filippo Maggi

**Affiliations:** 1 Department of Horticultural Science, School of Agriculture, Shiraz University, Shiraz 71441-65186, Iran; seyedsasanmousavi66@gmail.com (S.S.M.); tmovahhed@gmail.com (T.M.H.); 2 Department of Natural Resources and Environment Engineering, School of Agriculture, Shiraz University, Shiraz 71441-65186, Iran; saeedalizadehshz@gmail.com; 3 School of Pharmacy, University of Camerino, 62032 Camerino, Italy

**Keywords:** *Scrophularia striata*, allelochemicals, germination, ecotype, phenolic profile

## Abstract

A large number of plants produce secondary metabolites known as allelochemicals that are capable of inhibiting the germination of competitive species. This process is known as allelopathy and is mediated by several classes of chemicals, among which phenolic compounds are the most frequent. Thus, plant allelochemicals can be used to control weeds in agricultural systems. In the present work, we analyzed the phenolic profile and phytotoxic potential of different extracts (pure water or water: ethanol 50:50) from *Scrophularia* *striata* plants that were collected from two ecological regions in Iran (Pahleh and Lizan). The total polyphenolic content (TPC), as evaluated by the Folin-Ciocolteau method, ranged from 28.3 mg/g in the aqueous extract obtained from the Lizan ecotype to 39.6 mg/g in the hydroalcoholic extract obtained from the Pahleh ecotype. Moreover, HPLC analysis was aimed at determining the content of eight phenolic compounds, namely eugenol, rosmarinic acid, hesperetin, hesperedin, *trans*-ferulic acid, vanillin, and caffeic acid. According to the results, rosmarinic acid appeared to be the most abundant component. The phytotoxic activities of *S.* *striata* extracts were examined on the seed germination of a crop species, *Lepidium sativum*, and two weeds, *Chenopodium album* and *Malva sylvestris*. All extracts showed inhibitory effects on these species. The efficiency of these inhibitory effects depended on the type of plant species, origin, and concentration of extract. The highest phytotoxic activity was caused by approximately 1% concentration of extract. The most susceptible weed was *M. sylvestris*. The extracts that were obtained from the Pahleh ecotype, notably the hydroalcoholic ones, showed higher phytotoxicity against *L. sativum*, *C. album* and *M. sylvestris*. These results encourage further studies to support the use of *S. striata* as a source of bioherbicides.

## 1. Introduction

*Scrophularia striata* Boiss., known as “Tashaneh Dari” [1], belongs to the Scrophulariaceae family and grows widely in several regions throughout the world. Its main center of distribution is Iran [2]. Scrophulariaceae is a large angiosperm family, widely distributed in central Asia, Europe, and North America. It comprises about 3000 species and 220 genera [3,4]. The distribution of *S. striata* occurs predominantly in the arid and semi-arid regions of southwestern Iran [5]. Alkaloids, phenolics, iridoids, and cryptophilic acids are active constituents that accumulate in different parts of *S. striata* [6,7,8]. In the Iranian folk medicine, this species has been used as a wound healing agent [9].

Weeds are a significant threat to crop productivity in agroecosystems since the beginning of agriculture on earth, and the lack of weed control is a pressing concern among farmers [10]. Thus, herbicides have been mostly effective in controlling weeds [11]. Nonetheless, there is a growing concern among the public scientific community and farmers regarding the harmful impact of herbicide agrochemicals on the environment, whereby herbicide-resistant weeds can develop [12]. Allelopathy is a natural ecological function by which some organisms influence the activity of other organisms living in their surroundings. The influence is achieved by releasing secondary metabolites, also called allelochemicals [13,14,15]. The use of synthetic products is commonly discouraged due to their high cost and the ecological problems related to their usage. Scientists are trying to find new environmental solutions for plant growth enhancement that can ensure biosafety and eco-sustainability [16]. The utilization of allelopathic components as bioherbicides in agricultural systems can have many advantages over the usual, synthetic products [17,18]. Several compounds, including phenolics and their degradation products, were proven to have intense phytotoxic activity. Their significant inhibitory effects are seen on seed sprouting and disruption of root development [19]. Phenolic compounds are a major group of allelochemicals, ranging from phenols, flavonoids, hydroxycinnamic and benzoic acids, phenylpropanoids, coumarins, and tannins. They are produced by various plant species, while their inhibitory effects on crops and weeds have been well documented [12,20]. Allelochemicals are natural phytotoxins that offer a viable option for weed control in sustainable agroecosystems as an alternative to commercial herbicides. In the current study, the bioherbicidal potential of *S. striata* extracts were evaluated using an established method for allelopathic studies. The target plants were a crop species (*Lepidium sativum* L.) and two weed species (*Chenopodium album* L. and *Malva sylvestris* L.).

## 2. Results and Discussion

### 2.1. Total Phenol Content (TPC) of Different Ecotypes of Scrophularia striata

**Figure 1:**
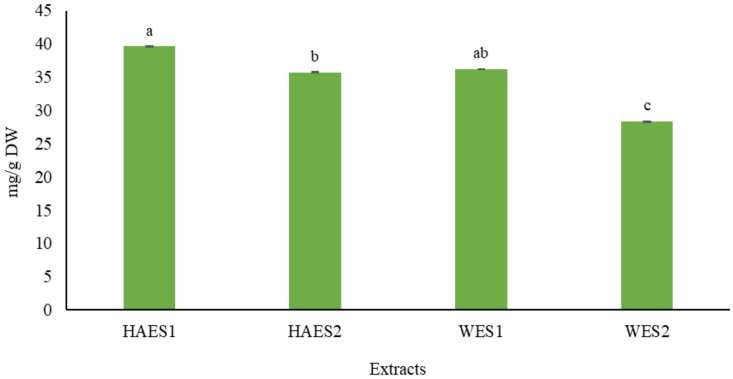
Total phenol content (TPC) (mg/g dry weight) of *Scrophularia striata* aerial parts extracts (HAES1: Hydroalcoholic extract Pahleh ecotype; HAES2: Hydroalcoholic extract Lizan ecotype; WES1: Water extract Pahleh ecotype; WES2: Water extract Lizan ecotype). Variables with different letters show statistically significant differences (*p* ≤ 0.05). The mean values with the same letters are not significantly different at 5% according to the LSD test.

The results showed that the TPC values of *S. striata* in water extracts (WE) and hydroalcoholic extracts (HAE) were significantly different in comparison to one another. The highest TPC value was observed in the HAE obtained from the Pahleh ecotype (39.6 mg/g DW), whereas the lowest one was obtained in the WE from the Lizan ecotype (28.3 mg/g DW) (Figure 1). This confirms that environmental conditions can influence the chemical characteristics of different ecotypes [21]. Indeed, variations in TPC reportedly exist in different ecotypes of legumes [22], *Camellia sinensis* (L.) Kuntze [23], *Prunus cerasus* L. [24], *Cistus monspeliensis* L. [25], and *Zataria multiflora* Boiss. [26]. Variations in quality and quantity of different secondary metabolites, including phenolics, under different extraction conditions, have been reported in olive pomace [27], *Moringa oleifera* Lam. [28], hazelnut skin [29], *Hamelia patens* Jacq. [30] and *Sideritis trojana* Bornm. [31]. Additionally, our results showed that the climatic conditions and the type of solvent can significantly affect the phytochemical composition of a plant extract. Previous research revealed the effects of different solvents on the phytochemical composition in extracts taken from different species, e.g., turmeric, lemon grass [32], *Momordica charantia* L. [33], kumquat [34], *Phoenix sylvestris* (L.) Roxb. fruit [35] and guava [36]. The quantity of polyphenols seems to be related to climatic variations. Actually, in warmer climates, phenolic compounds usually increase in amount, primarily due to the enhancement of photosynthetic activity [37,38]. Similar to our results, phytochemical variations have been observed in *Medicago minima* (L.) L. [39], *Vitis vinifera* L. [40], *Aloe vera* (L.) Burm.f. [41], *Thymus capitatus* (L.) Hoff. et Link. [42] and *Cichorium spinosum* L. [43], depending on geographic and climatic features.

### 2.2. HPLC Analysis of Phenolic Compounds in Scrophularia striata

**Figure 2:**
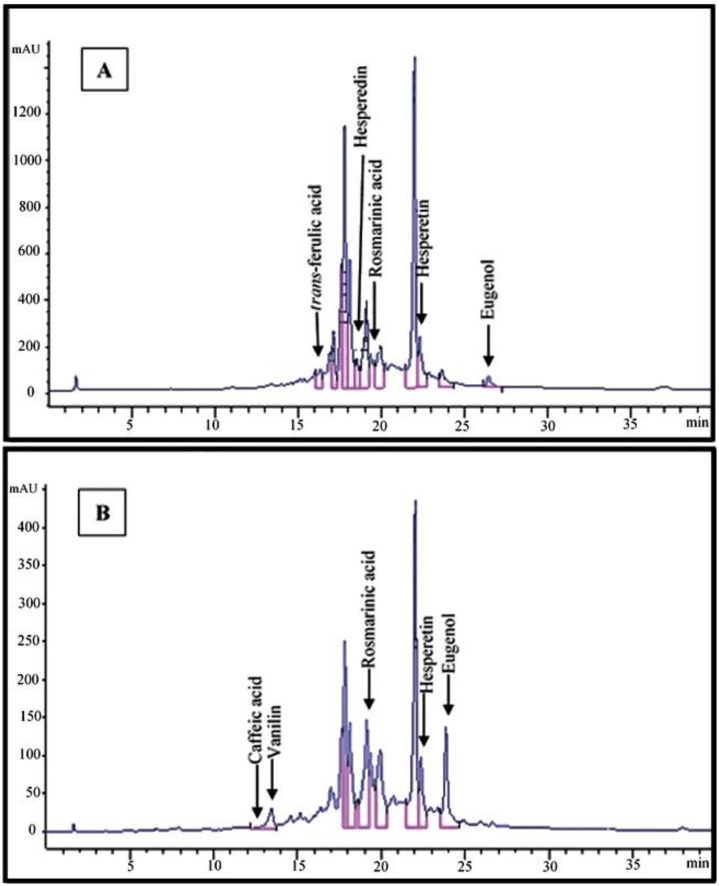
HPLC chromatograms of Pahleh extract (**A**) and Lizan extract (**B**) of *Scrophularia striata*.

HPLC analysis allowed us to determine quantitative values for seven phenolic compounds in the extracts of *S. striata* from the two ecotypes. The contents of vanillin (0.12 mg/g) and eugenol (2.12 mg/g) were higher in the Lizan extract, while rosmarinic acid (2.65 mg/g), hesperetin (1.27 mg/g), hesperedin (1.04 mg/g) and *trans*-ferulic acid (0.56 mg/g) were found in higher amounts in the Pahleh extract. Methanolic extracts were used for the preliminary identification of the target compounds in the two plant ecotypes. Caffeic acid was observed in trace amounts in the Lizan extract, while it appeared to be absent in the Pahleh extract (Table 1, Figure 2A,B). 

Chromatograms are also presented in Figure 2A,B. Phenolic compounds are the most commonly reported metabolites that play roles in defensive mechanisms in higher plants. With variable levels of toxicity, they target plant cellular functions at multiple sites [44]. A previous research indicated how caffeic acid derivatives from *Bellis perennis* L. have potential allelopathic capacities. There were variations based on inhibitory effects on certain plants (e.g., *Dactylis hispanica* Roth) and stimulatory effects on others (e.g., *Aegilops geniculate* Roth) when considering the growth of herbal species [45]. When testing the allelopathic potential of different plant extracts, it is important to set up the most profitable extraction method to obtain the best yield of phytotoxic compounds. Thus, optimum extract concentration and type should be defined when acting against different weed species [46]. The contents and composition of different groups of secondary metabolites such as phenolic compounds regularly depend on extrinsic and intrinsic factors. Variations in specific metabolites may stem from climatic and cultivation features, origin and genetic factors, plant density, ontogenetic phases and seasonal variations, as well as extraction and drying procedures [47,48,49]. When in small amounts, polyphenols can act positively on seed sprouting. Nonetheless, at high doses, they may influence various metabolic and morphogenic processes in herbs by inducing oxidative stress [50]. The most active compounds as bio-herbicides are secondary metabolites, e.g., rosmarinic acid, pelargonic acid, carvacrol, eugenol, etc., which are currently used in weed management. Rosmarinic acid is a widespread polyphenol in many species of the Lamiaceae family. Its potential has been investigated in a wide range of biological contexts. Allelopathic effects of hesperidin have been mentioned in previous reports [51,52]. 

The presence of vanillin in eggplant root exudates is reportedly regarded as an inhibitory effect and an obstacle to cropping [53]. It is known that vanillin has phytotoxic effects on goose grass (*Eleusine indica* L.), particularly on its plantlet growth. Furthermore, vanillin had strong inhibitory impacts on sprouting rates and the plantlet growth of lettuce and radish, but showed strong stimulatory effects on radix elongation in rice [54]. Ferulic and caffeic acids were measured in fresh aerial parts and roots of 10 cultivars of alfalfa. Bioassays on leaf litter leachates from all these cultivars showed variable degrees of inhibition on the seeding growth of *Festuca arundinacea* Schreb. and *Sorghum* x *sudanense* (Piper) Stapf. [55]. 

In a relevant study, the phytotoxic action of eugenol was examined on *Lactuca sativa* L., and the results showed a reduction in the number of normal plantlets, fresh weight, radix, and stem length, which might be due to the inhibitory effects on the cell cycle and the occurrence of chromosomal abnormalities [56]. Rosmarinic acid induced alterations in plasma membrane permeability in *Phaeodactylum tricornutum* Bohlin [57]. Eugenol was phytotoxic against a range of weeds such as *Taraxacum officinale* (L.) Weber ex F.H.Wigg. [58], *Amaranthus retroflexus* L. [59], *Chenopodium album* L. [60], *Echinochloa crus-galli* (L.) P.Beauv., *Phalaris minor* Retz., *Ageratum conyzoides* (L.) L., *Leptochloa chinensis* (L.) Nees, *Bidens Pilosa* L., and *Commelina benghalensis* L. [58], being promising as a bio-herbicide. 

### 2.3. Effects of Scrophularia striata Water Extracts on the Germination of Weeds

In the present study, the germination percentage of seeds was reduced significantly (*p* ≤ 0.05) in response to almost all extracts, as compared to the control. All concentrations of WE, which had been obtained from the Pahleh accession, could significantly reduce the germination percentage of the two weeds, *M. sylvestris* and *C. album* (*p ≤* 0.05). In the case of *M. sylvestris*, the first three concentrations reduced the germination percentage to zero. For *C. album*, however, the decrease was observed by up to 95%, similar to the control treatment. In *L. sativum,* the 0.25% concentration did not affect the germination, but the other two concentrations (0.5 and 0.75%) had stimulatory effects on the germination percentage of *L. sativum*. All concentrations of WE obtained from Lizan caused a significant decrease in seed germination (%) (*p ≤* 0.05) by up to 90–95% in *M. sylvestris* and *C. album*, whereas no significant decline was observed in the case of *L. sativum* germination (*p ≤* 0.05) in comparison with the control. In other words, Lizan WE promoted *L. sativum* germination (Table 2). 

A dose-dependent relationship was observed in regards to the inhibitory impact and elongation of radicle and stem. Previous research suggests that the disruption of mitochondrial respiration and oxidative pentose phosphate pathways are crucial mechanisms for the prevention of seed germination by phytochemicals [61]. Furthermore, phytotoxicity could be enhanced due to the synergistic impact of different phenolic compounds present in the extract [62].

A study on wheat showed that the foliar application of allelopathic WEs, and separately, seed pre-treatments in association with allelopathic WEs, can enhance grain weight and yield of plants, along with improvements in chlorophyll quantity under water-deficit stress. These observations can be explained by the accumulation of free proline in the leaf, total soluble phenolic compounds and a lower degree of relative membrane permeability in leaf cells [63]. In another research, the allelopathic activity was evaluated in aqueous extracts obtained from the roots and shoots of *Argemone ochroleuca* Sweet. Meanwhile, *Farsetia aegyptia* Turra, *Salvia aegyptiaca* L., *Hordeum vulgare* L., and *Medicago sativa* L. showed different responses to allelochemicals of *A. ochroleuca*. In a way, it could be said that the inhibition of seed germination is species-specific, while the concentration of extract, the type of plant organ and habitat conditions are likewise determinant. *F. aegyptia* seed germination is reportedly regarded as the most susceptible to different aqueous concentrations, whereas *H. vulgare* seed germination is the least susceptible [64]. 

In another research, the aqueous leaf extract of *Cymbopogon citratus* (DC.) showed allelopathic effects on the seed germination of *Bidens pilosa* L. and *B. subalternans* DC. The research revealed that the aqueous extract of *C. citratus* significantly reduced the seed germination rate of *B. pilosa* and *B. subalternans*. The concentrations interfered, inversely and proportionally, while reducing the germination rate [65]. A previous study was conducted on the phytotoxic potentials of aqueous leaf extracts obtained from *Artemisia absinthium* L. and *Psidium guajava* L. Evaluations were aimed at various effects on seed germination, plantlet development, enzymatic and non-enzymatic antioxidants, photosynthetic pigments, and osmolytes of *Parthenium hysterophorus* L. Leaf extracts of both *A. absinthium* and *P. guajava* acted to limit seed germination and plantlet development, but increased antioxidant enzyme activities [66].

### 2.4. Effects of Scrophularia striata Hydroalcoholic Extracts on the Germination of Weeds

All three species in the present study were significantly affected by Pahleh HAE (*p* ≤ 0.05). At high concentrations, germination was completely inhibited. On *L. sativum*, increasing the HAE Pahleh concentration caused a complete inhibition of germination. At a low concentration, Lizan HAE (0.25%) acted to stimulate germination in all three species, but higher concentrations (0.5–0.75 and 1%) caused a decrease of up to 90% in germination (Table 2). The inhibitory impact was found to be dose-dependent and intensified, in parallel to the increase in concentration of the aqueous leaf extracts [67]. Similar inhibitory effects were caused by aqueous leaf extracts of *Vitex negundo* L. on *Brassica chinensis* L., *L. sativa*, *Digitaria decumbens* Stent, and *Mimosa pudica* L. [68]. Raoof and Siddiqui [69] reported that aqueous extracts from the leaves and stems of *Tinospora cordifolia* (2 and 4%) significantly inhibited the seed germination and seedling growth of *C. album*, *C. murale* L., *Cassia tora* L., and *C. sophera* L. The inhibition intensified by increasing the concentrations of various aqueous extracts [70]. Polyphenols have phytotoxic properties that are enacted by disrupting vital processes during the germination phase. Polyphenols can inhibit seed germination by inducing oxidative stress and bringing restrictions on nutrient reserve mobilization. This is partly due to the inhibition of the activities of α-amylase and gibberellic acid [71].

The allelopathic activities of *Tectona grandis* L.f. [72], *Albizia procera* (Roxb.) Benth. [73], and *Acacia nilotica* (L.) Delile [74] were studied in regards to the sprouting and development of soybean. Accordingly, the leaf extracts of all these species at low doses had stimulatory effects on germination, growth, chlorophyll, protein, carbohydrates, and proline contents in soybean plants. At higher concentrations, however, there was a decrease in all of the measured attributes [75]. In some respects, the aforementioned research yielded similar results, thereby validating previous observations reported by Wang et al. [76], Khang et al. [77], Nouri et al. [78], and Al-Harbi, [79]. Allelochemicals have inhibitory effects and sometimes lethal impacts on seed germination, growth, and development of plants. According to these citations, lower concentrations prevent germination to various extents, which is probably due to the lower contents of allelochemicals, while higher concentrations cause lethal impacts on seed sprouting.

In a previous research, the impacts of several concentrations of a hydro-alcoholic extract of St. John’s wort (*Hypericum perforatum* L.) and sage (*Salvia officinalis* L.) were evaluated on seed sprouting (%) and germination rate of two weed species, *A. retroflexus* and *Portulaca oleracea* L. Their results revealed that the extract of St. John’s wort and sage had notable inhibitory effects on the sprouting of *A. retroflexus* seeds, but not on *P. oleracea* seeds [80,81]. In another research, the comparative allelopathic potentials of WE and HAE extracts of *Prangos ferulacea* (L.) Lindl. were studied on different plant organs (i.e., flower, stem, and leaf). The effects were investigated on the proline content, seed sprouting, and plantlet development of *Trifolium resupinatum* L. [82]. The results showed that the HAE extract showed maximum phenol and flavonoid contents, along with maximum phytotoxic impacts on *T. resupinatum*. Notably, it reduced seed sprouting and the plantlet development of *T. resupinatum*, while enhancing the proline content. These findings indicate that hydroalcoholic extracts can induce stronger oxidative stress on *T. resupinatum* [82].

### 2.5. Effects of Water Extracts Obtained from Scrophularia striata on the Root Length and Stem Length of Weeds

As shown in Table 2, the roots reached maximum length in the control treatment of all three species. However, root length decreased significantly (*p* ≤ 0.05) in all three examined species in response to all concentrations of WE from the Pahleh accession. The WE from the Lizan ecotype reduced significantly the root length in *M. sylvestris* and *C. album* (*p* ≤ 0.05). In *L. sativum*, low concentrations (0.25 and 0.5%) of Lizan WE did not reduce the root length, but higher concentrations were significantly effective (*p* ≤ 0.05) (Table 2). 

The stem length of some plants was affected significantly (*p* ≤ 0.05) at almost all concentrations. The Pahleh WE, at the three concentrations, could reduce *M. sylvestris* stem length significantly, although none of its concentrations could affect *C. album* stem length. The physiological response of *L. sativum* to Pahleh WE was also variable at different concentrations (Table 2). In one relevant study, the aqueous extracts of *Rhazya stricta* Decne. showed allelopathic activity while affecting *Salsola villosa* Schult. roots and stem length. More specifically, exudates from *R. stricta* leaves were observed to reduce the content of membrane polyunsaturated fatty acids and phospholipids, while hampering membrane functions and photosynthetic capacity. They also restricted the growth of *S. villosa* roots and stems [83]. Another study on aqueous leaf extracts of *Prosopis juliflora* (Sw.) DC. showed pronounced inhibitory effects on the root length of *Triticum aestivum* L. [84]. In another study, the WE of *Eclipta alba* (L.) leaves showed inhibitory effects on the root radicle length and stem length of *C. tora* and *C. sophera* [85]. An experiment was carried out to investigate the allelopathic activity of *T. cordifolia* extracts on plantlet development in *C. album*, *C. murale*, *C. tora*, and *C. sophera*. It was found that WE from the roots inhibited the growth of seedlings, roots, and shoots [69].

### 2.6. Effects of Scrophularia striata Hydroalcoholic Extracts on the Root and Stem Length of Weeds

All concentrations of HAE from the Pahleh ecotype significantly reduced the root length in all the examined species (*p ≤* 0.05). In *M. sylvestris*, the root length ceased to exist in response to all concentrations of the extracts. In *C. album*, high concentrations of HAE (0.5–0.75 and 1%) reduced the root length to zero. This happened almost similarly in the case of *L. sativum*, although the decrease was mostly in response to the 1% concentration of Pahleh HAE. However, Lizan HAE could not affect the root length of *M. sylvestris*, though it did reduce the root length of the two other plant species (*C. album* and *L. sativum*) at all concentrations. In applying the treatments on *C. album*, a more dramatic decrease in root length was observed as the concentration of Lizan HAE increased. Evaluations of the stem length under different treatments and concentrations showed that the Pahleh HAE reduced significantly the stem length in *M. sylvestris* and could totally prevent the stem growth. On *L. sativum*, two concentrations of this HAE (0.75 and 1%) reduced significantly the stem length to zero (*p* ≤ 0.05). The Lizan HAE extract, at 0.75 and 1% concentrations, reduced significantly the stem length in *M. sylvestris* and *L. sativum*, respectively (*p* ≤ 0.05) (Table 2). In a relevant study, the allelopathic potentials of hydroalcoholic foliar extracts of *Myrcia guianensis* (Aubl.) DC. were studied on the sorghum root growth. The results showed that HAE can have stronger inhibitory effects than methanol and aqueous extracts [86]. In another research, the allelopathic activity of *Mansoa standleyi* (Steyerm.) A.H.Gentry was investigated by comparing the impacts of the HAE on *M. pudica* roots, hypocotyl sprouting and development. It was observed that the HAE can inhibit the radicle growth and hypocotyl germination/development [87]. Researchers revealed that the HAE of *Withania somnifera* (L.) Dunal can have promising allelopathic properties by affecting significantly the seed germination and root elongation. *Cicer arietinum* L. and *T. aestivum* were mostly affected in a concentration-dependent manner by the *W. somnifera* extract [88]. Caffeic acid and ferulic acid, as phenolic compounds, notably reduced the root growth of mung bean and maize [89,90]. Compounds that can potentially induce inhibitory effects on seed germination and other growth factors are generally identified as phenolic acids. In this regard, *p*-coumaric, ferulic, vanillic and syringic acids are constituents of *V. negundo* leaf extracts. These compounds have shown both inhibitory and stimulatory effects on weeds, depending on the situation. Alterations in chlorophyll production, respiration, protein synthesis, hormonal balance and water content might be parameters influencing the allelopathic potential of extracts [91,92]. Abdelmigid and Morsi [93] reported that *Eucalyptus globulus* Labill. extract can reduce protein content in the root and stem of finger millet (*Eleusine coracana* (L.) Gaertn.). 

### 2.7. Effects of Scrophularia striata Water Extracts on the Fresh and Dry Weight of Weeds

Lizan WE reduced significantly the seedling fresh weight of *M. sylvestris* and *L. sativum* (*p ≤* 0.05) at a concentration of 0.5 and 1%, respectively. The WE of the Pahleh ecotype reduced the *M. sylvestris* fresh weight by 100% when used at concentrations of 0.25, 0.5, and 0.75%. Regarding *C. album* fresh weight, the highest degree of reduction (94%) occurred in response to the 0.75% concentration. Additionally, regarding *L. sativum* fresh weight, all concentrations of the extracts caused a significant decrease (*P ≤* 0.05), except for the 1% concentration (Table 2). Our results showed that the Pahleh extract can inhibit the germination and hamper the initial growth parameters more strongly in many cases, mostly because of its richer content in phenolic compounds such as *trans*-ferulic acid (0.56 mg/g), hesperedin (1.04 mg/g), hesperetin (1.27 mg/g), and rosmarinic acid (2.65 mg/g) (Table 1).

Dry weights of the different species followed nearly the same trend. Lizan WE reduced significantly the seedling dry weight of *C. album* at all concentrations (*p ≤* 0.05). Pahleh WE reduced the dry weight of all plants. These reductions were all significant (*p ≤* 0.05) considering *C. album* (Table 2). In almost all cases, high concentrations of the aqueous extracts reduced the fresh and dry weight of seedlings. Research on the allelopathic potential of aqueous extracts of peppermint revealed that the inhibitory impacts on tomato biomass occurred mostly in response to the application of 10% extract [71]. 

The aerial parts and root extracts of *Haloxylon aphyllum* (Minkw.) reduced the seedling fresh and dry biomass of *Agropyron elongatum* (Host.) and *A. desertorum* (Fisch.) [62]. Other researchers stated that the aqueous extracts of *T. cordifolia* (from the leaf and stems of the plant) reduced the dry weights of some weed plants [69]. In another study, *C. album* and *Coronopus didymus* (L.) Sm. fresh biomass was reduced by 100 and 90%, respectively, as a result of the allelopathic effects of sorghum (15 L/ha) [94]. In another investigation, sunflower aqueous extract (100% concentration) reduced the total dry weight of weeds, including *Avena fatua* L., *Phalaris minor* Retz., *C. album,* and *C. didimus* [95].

Low concentrations of benzoic, coumaric, gallic and caffeic acids can increase the cell division and cell enlargement by accelerating the rate of mitosis and cellulose synthesis [96], thereby providing an opportunity for a better growth of maize seedlings. An increase in protein content might also be due to the presence of phenolic compounds which help to assimilate the amino acids into structural protein-based building blocks in the plant [97]. Chlorogenic, isochlorogenic, neochlorogenic, and dicaffeoylquinic acids can be present in allelopathic extracts. They assist in preventing the production of ROS when applied at lower concentrations. In contrast, applying aqueous extracts that contain *p*-coumaric acid at low concentrations can strongly activate indolacetic acid (IAA). Cinnamic acid at low concentrations can prevent IAA from degradation and promote seedling growth. Phenolic compounds, having a variety of functions, act predominantly as antioxidant agents and reduce the harmful effects of reactive oxygen species [98,99].

### 2.8. Effects of Scrophularia striata Hydroalcoholic Extracts on the Fresh and Dry Weight of Weeds

In *L. sativum*, Lizan HAE caused a significant (*p ≤* 0.05) reduction in fresh weight in response to all concentrations. The Lizan HAE at 1% concentration reduced the fresh weight of *C. album* to zero. Furthermore, the HAE at 0.75 and 1% concentrations reduced the dry weight to zero in *M. sylvestris* and *C. album*, respectively. The HAE of the Pahleh ecotype reduced the *L. sativum* fresh weight significantly (*p ≤* 0.05) when used at concentrations of 0.5%, 0.75% and 1% (Table 2). In a relevant study, the results indicated how HAEs, which had been obtained from the leaf and stem of *Solidago gigantea* Aiton, affect the dry biomass of *E. crus-galli* and *A. retroflexus*. The decrease in dry biomass in these weeds depended on the concentration of extracts obtained from *S. gigantea*. The dry weights of *E. crus-galli* and *A. retroflexus* were reduced most notably by two concentrations, 12.5% and 10% [100]. Studies on *Protosiphom botryoides* Klebs-I. indicated that the growth rate and dry weight decrease in response to *Cestrum parqui* (Lam.) L’Hér. hydroalcoholic extract, especially at the lowest concentration [101]. Ravlić et al. [102] reported the inhibitory effects of fresh and dry biomass of scentless mayweed on the germination of cereals. Variations in the diffusion pattern of soluble allelochemicals during extraction can be a reason for differences in the inhibitory effects of extracts [103].

## 3. Materials and Methods 

### 3.1. Plant Collection Area

**Figure 3:**
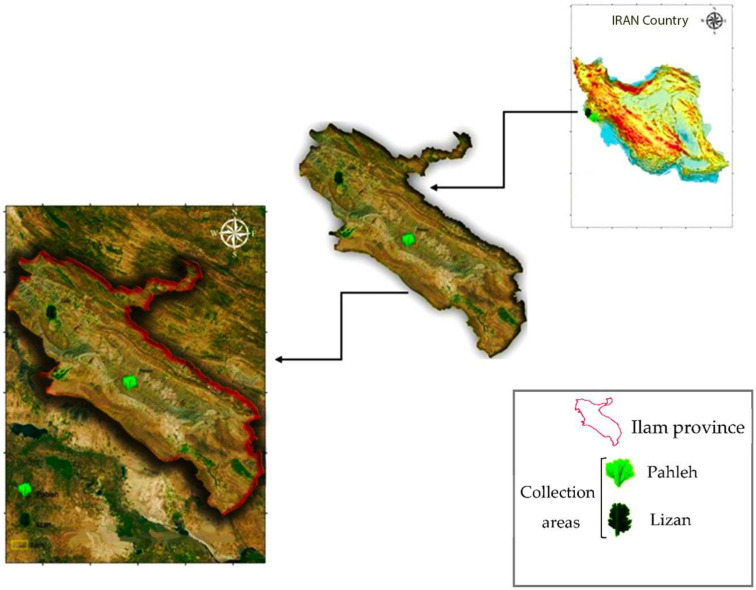
Different sampling locations of *Scrophularia striata* from Ilam province (the two sampling regions, Pahleh and Lizan, are shown in the map).

*S. striata* is mostly distributed in the province of Ilam, a mountainous region in the western part of Iran [104]. For the purpose of sampling, habitats of the herb were selected via field observations. Subsequently, two distinct regions were selected based on ecological factors. These regions were, namely, Lizan (Longitude: 46°8′19.42″ E; Latitude: 33°34′2.16″ N) and Pahleh (Longitude: 46°50′43.16″ E; Latitude: 33°2′27.28″ N), with a distance of 149 km apart from each other. The location of the studied sites is provided in Figure 3.

### 3.2. Plant Material

Aerial parts of *S. striata* were collected from two regions of Ilam province, namely, Pahleh (S1) and Lizan (S2). Sampling was carried out in May 2019. Aerial parts that were in the fruit set stage were used for extraction and phytochemical investigations. The plant materials were dried in the room temperature for one week. The seeds of two universally aggressive agricultural weeds (*Malva sylvestris* L. and *Chenopodium album* L.) were collected from the Agricultural Research Station of Shiraz University. Additionally, *L. sativum* seeds were bought from the market. As a widespread crop species, *L. sativum* is a leafy edible plant and is commonly used as a test species for initial screening in laboratory bioassays aimed at evaluating the phytotoxic effects of allelochemicals and/or natural phytotoxins [105].

### 3.3. Preparation of Aqueous and Hydroalcoholic Extracts for Allelopathy Experiments

To make the required extracts, the reflux apparatus was used according to the relevant literature and methodology, with specific changes [106]. Briefly, the dried and powdered aerial parts of *S. striata* (50 g from Pahleh ecotype and 50 g from Lizan ecotype) were used separately to make a stock aqueous extract (WE) solution. Additionally, 50 g dry aerial parts of the Pahleh ecotype and 50 g dry aerial parts of the Lizan ecotype were added to a mixture of water: ethanol with 96% purity (50:50) (500 mL). These were collectively placed in a round-bottomed laboratory balloon (1000 mL) to prepare the hydroalcoholic extract (HAE). Then, the balloons were placed in heating mantles, and the extraction process took 2 h. All assays were performed in triplicate. Filtrates of stock extracts were obtained by filter paper (Whatman No. 2) and were appropriately diluted to give final concentrations of 0.25, 0.5, 0.75, and 1% (*v*/*v*). Distilled water and water-ethanol mixtures were used as the control.

### 3.4. Extract Preparation for Phenolic Compounds Determination

The dry aerial parts of the two mentioned ecotypes were powdered. For each ecotype, 2 mL of solvent (85% methanol + 15% acetic acid) was added to 0.2 g of powder. The tubes were filled with nitrogen gas to prevent the oxidation of the phenolic compounds. They were settled at −20 °C in the dark for 24 h. After one day, the tubes were put in an ultrasonic bath for 15 min. Then, the tubes were centrifuged for 20 min (4 °C, 10,000 rpm), the supernatant was separated, and *n*-hexane (which occurred in the same volume as the supernatant) was added. Then, the tubes were vortexed for 15 secs and centrifuged again for 10 min (10,000 rpm, 4 °C). At this stage, the underside phase consisted of polyphenols, which can be separated by a syringe. A syringe filter (0.45 Micron) was used to remove impurities. Extracts were kept in special vials in the refrigerator until HPLC analysis.

### 3.5. Determining the Total Phenol and Phenolic Compounds Profile

The total phenolic content (TPC) of the aqueous and hydroalcoholic extracts were determined using the method of Mahdavikia and Saharkhiz [71]. Briefly, the calibration curve was based on a mixture, including 1 mL ethanolic solution of gallic acid (0.025–0.400 mg/mL), 5 mL diluted Folin-Ciocalteu reagent, and 4 mL sodium carbonate (0.7 M). The absorbance of TPC was measured by using a Hitachi U-2000 spectrophotometer at 765 nm. For separation, identification, and quantification of the phenolic components of *S. striata* extracts, an HPLC analysis was carried out on an Agilent 1200 series (USA), equipped with a reverse phase Zorbax Eclipse (XDB)-C18 column (10 cm × 5 mm i.d.; 150 mm film thickness), and with a photodiode array detector (PAD). 

To prepare the injectable extracts, 0.02 g of the vacuum-dried residue of the plant extract was dissolved in 1 mL of methanol. The aliquots were filtered through a 0.2 µm membrane millipore chromatographic filter and then 20 µL of the solution was injected into the HPLC system. The flow rate was set at 1 mL min^−1^. The elution was monitored at 280 and 320 nm. A gradient elution was selected to achieve maximum separation and sensitivity. The elution was performed by changing the proportion of solvent A (formic acid 1% in deionized water) to solvent B (methanol (*v*/*v*)) as follows: methanol: formic acid 1% (10:90), at 0 min; methanol: formic acid 1% (25:75), at 10 min; methanol: formic acid 1% (60:40), at 20 min and, finally, methanol: formic acid 1% (70:30), at 30 min. The total running time was 40 min. The column temperature was 30°C. Compounds were identified by comparison with analytical standards and by drawing calibration curves. The analytical standards were caffeic acid, vanillin, *trans*-ferulic acid, hesperedin, eugenol, hesperetin, and rosmarinic acid. All were purchased from Sigma.

### 3.6. Germination Bioassay

The phytotoxic effects of the two types of extracts, which had been obtained from two different ecotypes, Pahleh and Lizan, were evaluated. These effects were monitored on the crop plant and compared to the effects on weed species. Sprouting assessments were carried out for each *S. striata* extract as follows. Concerning seed size, 20 sterilized *M. sylvestris*, *C. album*, and *L. sativum* seeds were placed on Whatman No. 1 filter paper in sterile Petri dishes (9 cm diameter). Five ml of the extract solutions from four concentrations of stock solution (0.25, 0.5, 0.75, and 1.0%) were included in each plate. Distilled water and hydroalcoholic solution (ethanol 50%) were used as the control. The plates were placed in a controlled chamber (1300 STC Mod, Noor-Sanat-Ferdows Company, Karaj, Iran) (26 °C during the day and 22 °C at night, 16 h light and 8 h dark, 4000 lux) and germination tests took up to about two weeks. To stop evaporation, the plates were closed up with parafilm and then placed in the growth chamber. The plates were examined every day and moistened as required. Three replications were used for each treatment. After about two weeks, the seeds that showed radix unfolding (1 mm) were noted as sprouted. The final germination percentage (FGP) was calculated for each trial according to a method used by Mouradi et al. [107]. Moreover, the length of the seedlings primary radix and the stem length were measured by a ruler. Dry and fresh weights were measured at the end of the test with a four-digit scale.

### 3.7. Statistical Analysis

The investigation was arranged based on a completely randomized factorial design with three replications for each treatment. The treatments were two types of *S. striata* extracts (WE and HAE extracts) and three different plant species (*L. sativum, C. album*, and *M. sylvestris*). Each extract had four concentrations. Distilled water and a water-ethanol solution (50:50) were used as control groups. These treatments were assumed to affect the germination percentage and several other factors of seedling growth. The normality test and data analyzing were carried out by using Minitab statistical software (version17) to check data normality. The data, as mean values, were compared by using Tukey’s analyzed test at the 5% level.

## 4. Conclusions

The allelopathic effects of the aqueous and hydroalcoholic extracts of *S. striata* were documented after being obtained from two different ecotypes (Pahleh and Lizan). Inhibitory effects on seed germination and seedling growth were observed in *L. sativum, C. album,* and *M. sylvestris*. High concentrations of extracts yielded the best results but, in many cases, acted in a species-specific manner. The extracts of *S. striata* could have allelopathic effects on other weeds and horticultural plants because they are capable of substantial effects on weeds, thereby having the capacity to contribute to an environmentally friendly approach to weed control. These extracts deserve further tests on other weeds. Different variations in the components of *S. striata* extracts can be valuable from a phytotoxic point of view to formulate natural herbicides. More research is required in this regard to operationalize in agriculture the allelochemicals that are currently identified in hydroalcoholic extracts obtained from *S. striata*. Indeed, the evaluation of their effects against weed species under field conditions can support their use as natural herbicides.

## Figures and Tables

**Table 1:** Polyphenol profile, as determined by HPLC in the two *Scrophularia striata* ecotypes.

Compound (mg/g)	Lizan	Pahleh
Caffeic acid	+	ND
Vanillin	0.12 ± 0.01	ND
*trans*-Ferulic acid	ND	0.56 ± 0.05
Hesperedin	ND	1.04 ± 0.04
Eugenol	2.12 ± 0.06	1.88 ± 0.03
Hesperetin	0.53 ± 0.02	1.27 ± 0.02
Rosmarinic acid	0.88 ± 0.04	2.65 ± 0.04

+ means that this compound was detected in the extract but its content was very low (<limit of quantification); ND, not detected.

**Table 2:** Different concentrations of aqueous and hydroalcoholic extracts obtained from *Scrophularia striata*. The effects of these extracts on germination and several growth parameters of the examined species are shown. Mean values followed by the same letter are not significantly different at *p* ≤ 0.05 (Tukey’s test).

Concentration	Treatment	Germination Percentage (%)			Root Length (cm)			Stem Length (cm)			Fresh Weight (mg)			Dry Weight (mg)		
		*M. sylvestris*	*C. album*	*L. sativum*	*M. sylvestris*	*C. Album*	*L. sativum*	*M. sylvestris*	*C. album*	*L. sativum*	*M. sylvestris*	*C. album*	*L. sativum*	*M. sylvestris*	*C. album*	*L. sativum*
0	Control-WE	53.33 ± 5.8 ^de^	63.33 ± 5.8 ^cd^	86.67 ± 5.8 ^ab^	3.63 ± 0.25 ^b^	5.47 ± 0.66 ^ab^	7.25 ± 0.28 ^a^	4.43 ± 0.47 ^ab^	3.23 ± 0.62 ^ab^	5.21 ± 0.36 ^a^	1100 ± 205.02 ^ab^	870 ± 37.86 ^a−c^	1410 ± 305.67 ^a^	2.67 ± 1.15 ^b^	20 ± 1.53 ^a^	4.33 ± 2.31 ^b^
0.25	Pahleh WE	0 ± 0 ^l^	8.33 ± 5.8 ^h–l^	86.67 ± 12.6 ^ab^	0 ± 0 ^g^	2.51 ± 1.07 ^b–d^	2.28 ± 0.54 ^b–f^	0 ± 0 ^f^	2.65 ± 1.0 ^bc^	2.36 ± 0.95 ^b–e^	0 ± 0 ^f^	630 ± 160 ^b–f^	520 ± 30.55 ^b–f^	0 ± 0 ^b^	3.67 ± 2.08 ^b^	1.67 ± 0.58 ^b^
0.5		0 ± 0 ^l^	3.33 ± 2.9 ^j–l^	93.33 ± 2.9 ^a^	0 ± 0 ^g^	1.34 ± 1.16 ^c–g^	2.02 ± 0.31 ^b–g^	0 ± 0 ^f^	1.95 ± 1.69 ^b–f^	2.33 ± 0.66 ^b–f^	0 ± 0 ^f^	470 ± 430.62 ^b–f^	660 ± 275.38 ^b–f^	0 ± 0 ^b^	4.33 ± 3.78 ^b^	2.67 ± 2.08 ^b^
0.75		0 ± 0 ^l^	6.67 ± 2.9 ^i–l^	95.00 ± 5.0 ^a^	0 ± 0 ^g^	0.60 ± 0.2 ^d–g^	1.85 ± 0.40 ^b–g^	0 ± 0 ^f^	1.35 ± 0.54 ^b–f^	1.94 ± 0.71 ^b–f^	0 ± 0 ^f^	50 ± 20 ^ef^	580 ± 55.07 ^b–f^	0 ± 0 ^b^	0.3 ± 0.17 ^b^	1.67 ± 0.58 ^b^
1		10.00 ± 17.3 ^h–l^	3.33 ± 2.9 ^j–l^	73.33 ± 10.4 ^bc^	0.34 ± 0.59 ^d–g^	1.37 ± 1.18 ^c–g^	1.47 ± 0.56 ^c–g^	0.54 ± 0.94 ^b–f^	1.02 ± 0.88 ^b–f^	2.73 ± 0.92 ^ab^	160 ± 282.90 ^d–f^	320 ± 280 ^b–f^	720 ± 160.73 ^a–e^	0.67 ± 1.15 ^b^	1.67 ± 1.52 ^b^	3.33 ± 1.15 ^b^
0.25	Lizan WE	21.67 ± 2.9 ^g–j^	13.33 ± 2.9 ^h–l^	90.00 ± 10.0 ^ab^	0.48 ± 0.15 ^d–g^	1.51 ± 0.31 ^c–g^	7.43 ± 0.02 ^a^	1.67 ± 0.53 ^b–f^	1.43 ± 0.61 ^b–f^	2.50 ± 0.11 ^b–d^	400 ± 162.89 ^b–f^	510 ± 109.70 ^b–f^	1150 ± 45.83 ^a^	2.07 ± 2.57 ^b^	1.43 ± 1.40 ^b^	90 ± 0.58 ^b^
0.5		6.67 ± 2.9 ^i–l^	11.67 ± 2.9 ^h–l^	88.33 ± 7.6 ^ab^	0.16 ± 0.04 ^fg^	2.00 ± 0.98 ^b–g^	6.47 ± 1.40 ^a^	0.09 ± 0.005 ^ef^	2.83 ± 0.86 ^ab^	2.00 ± 0.46 ^bf^	10 ± 0.63 ^f^	760 ± 283.61 ^b–d^	1140 ± 115.33 ^a^	0.1 ± 0.1 ^b^	3 ± 1.73 ^b^	80 ± 0.58 ^b^
0.75		3.33 ± 2.9 ^j–l^	6.67 ± 2.9 ^i–l^	93.33 ± 7.6 ^a^	0.87 ± 1.03 ^cg^	2.84 ± 0.05 ^bc^	3.80 ± 1.03 ^b^	1.73 ± 1.58 ^bf^	2.09 ± 0.35 ^bf^	1.99 ± 0.68 ^b–f^	360 ± 558.24 ^b–f^	590 ± 219.32 ^b–f^	870 ± 70.24 ^a–c^	2.67 ± 3.79 ^b^	1.23 ± 1.53 ^b^	70 ± 0.58 ^b^
1		3.33 ± 2.9 ^j–l^	6.67 ± 2.9 ^i–l^	95.00 ± 8.7 ^a^	1.43 ± 1.25 ^c–g^	1.12 ± 0.90 ^c–g^	3.80 ± 1.31 ^b^	2.03 ± 1.76 ^b–f^	2.63 ± 0.61 ^a–c^	2.50 ± 0.10 ^b–d^	590 ± 516.43 ^b–f^	610 ± 220.30 ^b–f^	670 ± 111.35 ^b–f^	4 ± 3.60 ^b^	2.67 ± 2.08 ^b^	3.67 ± 0.58 ^b^
0	Control-HAE	20.00 ± 5.0 ^h–k^	36.67 ± 2.9 ^e–g^	70.00 ± 5.0 ^bc^	2.267 ± 0.45 ^b–f^	4.28 ± 0.54 ^b^	6.27 ± 0.34 ^a^	2.40 ± 0.17 ^bd^	2.16 ± 0.15 ^bf^	3.68 ± 0.09 ^ab^	560 ± 212.21 ^af^	300 ± 20.82 ^b–f^	1070 ± 66.58 ^a^	3 ± 1.73 ^b^	2.03 ± 0.91 ^b^	8.4 ± 1.04 ^b^
0.25	Pahleh HAE	0 ± 0 ^l^	6.67 ± 2.9 ^i–l^	66.67 ± 2.9 ^cd^	0 ± 0 ^g^	0.60 ± 0.1 ^d–g^	3.72 ± 0.13 ^b^	0 ± 0 ^f^	0.41 ± 0.10 ^b–f^	2.88 ± 0.18 ^ab^	0 ± 0 ^f^	3.33 ± 2.08 ^f^	960 ± 40 ^ab^	0 ± 0 ^b^	0.2 ± 0.1 ^b^	6 ± 1 ^b^
0.5		0 ± 0 ^l^	0 ± 0 ^l^	51.67 ± 12.6 ^de^	0 ± 0 ^g^	0 ± 0 ^g^	2.02 ± 1.47 ^b–g^	0 ± 0 ^f^	0 ± 0 ^f^	2.14 ± 1.60 ^b–f^	0 ± 0 ^f^	0 ± 0 ^f^	540 ± 471.03 ^b–f^	0 ± 0 ^b^	0 ± 0 ^b^	3.67 ± 2.31 ^b^
0.75		0 ± 0 ^l^	0 ± 0 ^l^	8.33 ± 5.8 ^h–l^	0 ± 0 ^g^	0 ± 0 ^g^	0 ± 0.08 ^fg^	0 ± 0 ^f^	0 ± 0 ^f^	0 ± 0.09 ^ef^	0 ± 0 ^f^	0 ± 0 ^f^	1.67 ± 1.15 ^f^	0 ± 0 ^b^	0 ± 0 ^b^	0.1 ± 0 ^b^
1		0 ± 0 ^l^	0 ± 0 ^l^	0 ± 0 ^l^	0 ± 0 ^g^	0 ± 0 ^g^	0 ± 0 ^g^	0 ± 0 ^f^	0 ± 0 ^f^	0 ± 0 ^f^	0 ± 0 ^f^	0 ± 0 ^f^	0 ± 0 ^f^	0 ± 0 ^b^	0 ± 0 ^b^	0 ± 0 ^b^
0.25	Lizan HAE	26.67 ± 5.8 ^f–h^	43.33 ± 5.8 ^ef^	76.67 ± 2.9 ^a–c^	0.66 ± 0.19 ^c–g^	0.80 ± 0.35 ^c–g^	2.38 ± 0.24 ^b–e^	0.34 ± 0.06 ^c–f^	2.27 ± 0.21 ^b–f^	2.25 ± 0.40 ^b–f^	290 ± 100.17 ^b–f^	60 ± 15.82 ^ef^	770 ± 45.09 ^b–d^	3.23 ± 2.25 ^b^	1.33 ± 0.58 ^b^	6.67 ± 1.15 ^b^
0.5		16.67 ± 5.8 ^h–l^	40.00 ± 10.0 ^e–g^	46.67 ± 5.8 ^e^	0.51 ± 0.20 ^d–g^	0.78 ± 0.11 ^c–g^	0.48 ± 0.17 ^d–g^	0.24 ± 0.13 ^d–f^	0.52 ± 0.10 ^b–f^	2.71 ± 0.49 ^ab^	60 ± 51.12 ^ef^	3.8 ± 1.06 ^f^	640 ± 81.44 ^b–f^	0.23 ± 0.15 ^b^	0.27 ± 0.11 ^b^	3.57 ± 2.50 ^b^
0.75		5.00 ± 5.0 ^i–l^	20.00 ± 0 ^h–k^	23.33 ± 5.8 ^g–i^	0.14 ± 0.16 ^fg^	0.42 ± 0.06 ^d–g^	1.38 ± 1.43 ^c–g^	0.07 ± 0.06 ^ef^	0.25 ± 0.08 ^df^	1.44 ± 1.19 ^b–f^	1.43 ± 2.23 ^f^	0.73 ± 0.30 ^f^	680 ± 581.74 ^bf^	0.1 ± 0.1 ^b^	0.27 ± 0.21 ^b^	30 ± 36.06 ^a^
1		1.67 ± 2.9 ^kl^	6.67 ± 2.9 ^i–l^	6.67 ± 5.8 ^i–l^	0.53 ± 0.92 ^d–g^	0.11 ± 0.02 ^fg^	0.25 ± 0.22 ^e–g^	0.90 ± 1.56 ^b–f^	0.03 ± 0.06 ^ef^	0.17 ± 0.15 ^d–f^	210 ± 369.50 ^c–f^	0.23 ± 0.11 ^f^	50 ± 42.10 ^ef^	1 ± 1.73 ^b^	0.1 ± 0 ^b^	0.07 ± 0.11 ^b^

Different letters show significant statistical difference (*p* ≤ 0.05). Three concentrations include 0.25, 0.5 and 0.75 were made from the stock solution (100% = 1).

## Data Availability

Not applicable.
